# Novel *in vitro* evidence on the beneficial effect of quercetin treatment in vascular calcification

**DOI:** 10.3389/fphar.2024.1330374

**Published:** 2024-01-26

**Authors:** E. Ceccherini, I. Gisone, E. Persiani, C. Ippolito, A. Falleni, A. Cecchettini, F. Vozzi

**Affiliations:** ^1^ Institute of Clinical Physiology, National Research Council, Pisa, Italy; ^2^ Department of Clinical and Experimental Medicine, University of Pisa, Pisa, Italy

**Keywords:** nutraceuticals, quercetin, vascular calcification, VSMCs, inflammation, *in vitro* model

## Abstract

Vascular calcification is a pathological chronic condition characterized by calcium crystal deposition in the vessel wall and is a recurring event in atherosclerosis, chronic kidney disease, and diabetes. The lack of effective therapeutic treatments opened the research to natural products, which have shown promising potential in inhibiting the pathological process in different experimental models. This study investigated the anti-calcifying effects of Quercetin and Berberine extracts on vascular smooth muscle cells (VSMCs) treated with an inorganic phosphate solution for 7 days. Quercetin has shown the highest anti-calcifying activity, as revealed by the intracellular quantitative assay and morphological analysis. Confocal microscopy revealed downregulation of RUNX2, a key marker for calcified phenotype, which was otherwise upregulated in calcified VSMCs. To investigate the anti-inflammatory activity of Quercetin, culture media were subjected to immunometric assays to quantify the levels of IL-6 and TNF-α, and the caspase-1 activity. As expected, calcified VSMCs released a large quantity of inflammatory mediators, significantly decreasing in the presence of Quercetin. In summary, our findings suggest that Quercetin counteracted calcification by attenuating the VSMC pathological phenotypic switch and reducing the inflammatory response. In our opinion, these preliminary *in vitro* findings could be the starting point for further investigations into the beneficial effects of Quercetin dietary supplementation against vascular calcification.

## 1 Introduction

Vascular calcification (VC) is a pathological condition characterized by the deposition of calcium-phosphate crystals in the inner and medial layers of the valve and vessel wall (Lee et al., 2020). Although VC is a pathological process related to aging (Pescatore et al., 2019), many other chronic diseases, such as atherosclerosis, diabetes mellitus, hypertension, and chronic kidney disease, are closely associated with VC occurrence (Palit and Kendrick, 2014; Nicoll et al., 2016; Grootaert et al., 2018; Giha et al., 2022). Multiple mechanisms have been proposed to explain the calcification within the vessel wall, including the osteogenic differentiation of vascular smooth muscle cells (VSMCs) (Ceccherini et al., 2022). In healthy vessels, these contracting cells remain quiescent, regulating the vascular tone and blood pressure. Several external stimuli, such as the imbalance of calcium-phosphate homeostasis, drive the VSMC phenotypical switch into osteoblast-like cells, characterized by the expression of specific osteochondrogenic markers (i.e., osteocalcin (OCN), alkaline phosphatase (ALP), and osteopontin (OPN), runt-related transcription factor 2 (RUNX2)) and loss of contractile markers expression (i.e., α smooth muscle actin). The osteoblast-like phenotype, also called “calcified,” is responsible for the active intracellular calcium deposition and extracellular matrix mineralization (Demer and Tintut, 2008). The lack of effective treatments has pushed research efforts towards natural extracts-derived molecules such as Quercetin (Anand David et al., 2016; Ceccherini et al., 2020; Aghababaei and Hadidi, 2023) and Berberine (Ai et al., 2021; Li et al., 2023) for their anti-inflammatory and antioxidant activity exerted in a broad variety of human pathologies, including cardiovascular diseases. Berberine is an isoquinoline alkaloid possessing ameliorative effects in experimental mouse models of VC by activating the Akt signaling pathway, and inhibiting either endoplasmic reticulum stress or inflammatory reaction (Li et al., 2022; Xiao et al., 2023). Berberine also preserved arterial elasticity in spontaneously hypertensive rats by increasing elastin fiber content (Zhang et al., 2020). Quercetin is a bioflavonoid belonging to polyphenols contained in many fruits and vegetables, and attracted attention for the variety of its cardiovascular-related benefits in humans and animal models, including the regulation of lipid metabolism, the anti-platelet aggregation, and the vasoconstriction (Patel et al., 2018; Papakyriakopoulou et al., 2022). However, limited *in vitro* studies have investigated its potential activity on vascular cell calcification (Beazley et al., 2013; Cui et al., 2017). Recent pieces of evidence indicated that Quercetin could affect the osteogenic switch of VSMCs, favoring the contractile phenotype. In particular, it would appear that Quercetin induced the downregulation of several markers related to calcified phenotype (i.e., β-catenin, Msh Homeobox 2 (Msx2), Bone Morphogenetic Protein 2 (BMP2), and Osterix) and upregulation of contractile proteins smooth muscle actin (SMA) and smooth muscle protein 22-alpha (SM22a) (Beazley et al., 2013; Liang et al., 2018). VSMC apoptosis is a cellular event that drives the calcification process, and derived-apoptotic bodies could act as nucleating nodes for calcium crystal formation (Proudfoot et al., 2000). Cui and colleagues highlighted the anti-apoptotic activity of Quercetin in calcified VSMCs by inhibiting the oxidative stress cascade and restoring proper mitochondrial activity (Cui et al., 2017). These preliminary data are encouraging and have pointed out the beneficial effects of Quercetin that could be useful in VC; therefore, in this study, we evaluated the anti-calcifying ability of 2 selected natural extracts, among which Quercetin was found to possess the highest potential. To elucidate the underlying mechanisms, markers related to calcified phenotype (runx2 and galectin-3) and inflammatory environment (interleukin-6 (IL-6), Tumor necrosis factor-α (TNF-α), and caspase-1) were also evaluated using confocal microscopy and immunometric assay.

## 2 Materials and methods

### 2.1 Cell cultures and phosphate-induced VSMC calcification

Human coronary artery smooth muscle cells (HCASMC, Lonza), hereafter abbreviated as VSMCs, were cultured in Medium 231 with Smooth Muscle Growth Supplement (Lonza) and Penicillin/Streptomycin for a final concentration of 100 I.U./ml and 100 μg/mL, respectively. Cells were cultured at 37°C, 5% CO_2,_ and kept at low passages. To induce calcification, VSMCs were treated with a calcification medium composed of 1.9 mM NaH_2_PO_4_/Na_2_HPO_4_ (1:1) in DMEM high glucose (Holmar et al., 2020) for 7 days, replacing the medium following 72 h treatment.

### 2.2 Cytotoxicity of natural extracts

Quercetin (purity of 98.1%) and Berberine (purity of 97.2%) extracts were dissolved in DMSO to obtain a 50 mM stock solution for Quercetin and 25 mM for Berberine. These solutions were stored at −20°C until use. In each experiment, stock solutions were diluted 1:10 in the culture medium and further diluted to reach the final concentration required. To assess the cytotoxic effects, 1,000 VSMCs were seeded in a 96-well plate and treated with the natural extracts in a range of 1 and 500 μM, replacing the medium following 72 h treatment. The viability test was performed following 7 days of treatments using the CellTiter-Blue^®^ Cell Viability Assay kit (Promega), according to the manufacturer’s instructions, measuring the fluorescence at 560/590 nm (Fernandes et al., 2023).

### 2.3 Anti-calcifying properties of natural extracts

The nutraceutical concentrations showing viability higher than 70% (according to EN ISO 10993-5) were selected to evaluate their anticalcifying properties. 1,000 VSMCs were seeded in a 96-well plate and treated for 7 days with the calcification medium supplemented with natural extracts, replacing the medium following 72 h treatment. At the end of the experiments, VSMC were washed twice with PBS, lysed by HCl 0.6 M treatment for 1 h at 4°C, and overnight at −20°C to complete the cell lysis. According to the manufacturer’s instructions, the intracellular calcium content was determined colorimetrically using the Calcium Colorimetric Assay Kit (Sigma), measuring the absorbance at 575 nm.

### 2.4 IL-6 quantification

In the culture media, the IL-6 levels were determined using a non-competitive chemiluminescent immunoassay (Roche Elecsys IL-6 Kit), according to the manufacturer’s protocol.

### 2.5 Caspase-1 activity

The activity of caspase-1 was determined using the Caspase-Glo^®^ 1 Inflammasome Assay (Promega) according to the manufacturer’s protocol. Briefly, 50 μL of culture medium was treated with the same volume of Caspase-Glo^®^ 1 Reagent and incubated at room temperature for 1 h. The luminescence was measured using a plate-reading luminometer.

### 2.6 TNF-α quantification

TNF-α Human ELISA Kit (Thermo Fisher) was used for TNF-α detection. Briefly, 50 μL of culture medium was incubated in a 96-well plate containing immobilized monoclonal TNF-α antibodies. Afterward, the biotin-conjugated anti-TNF–α and the streptavidin–horseradish peroxidase were added to each well. After that, the antibody–protein complex was detected by adding tetramethylbenzidine and measuring the absorbance at 450 nm.

### 2.7 Transmission electron microscopy

Following 7 days treatment with calcifying medium added to Quercetin 100 μM and calcifying medium alone, VSMCs were recovered using trypsin and centrifuged at 300 *g* for 5 min. VMSC pellets were fixed in 2.5% glutaraldehyde in 0.1 M cacodylate buffer, pH 7.2, for 2 h at 4°C and postfixed in 1% osmium tetroxide in the same buffer for 1 h at room temperature. Cells were then dehydrated in a graduated series of ethanol, embedded in Epon-Araldite, and polymerized at 60°C. Ultrathin sections (60–90 nm), obtained with a Reichert-Jung Ultracut E (Reichert-Yung, Wien, Austria) equipped with a diamond knife, were collected on 200-mesh formvar/carbon-coated copper grids, double stained with aqueous uranyl acetate and lead citrate, and examined with a Jeol 100 SX Transmission electron microscope (Jeol, Tokyo, Japan) operating at 80 kV. Micrographs were obtained with an AMTXR80b Camera System.

### 2.8 Confocal microscopy

20,000 VSMCs were seeded on a 6-well glass coverslip and treated for 7 days with the calcification medium supplemented with natural extracts, replacing the medium following 72 h treatment. At the end of experiments, VSMC were washed twice with PBS, fixed with 4% paraformaldehyde for 30 min at 4°C, and permeabilized with 0.1% Triton X-100 (diluted in PBS) for 10 min at room temperature. A blocking solution containing 1% BSA and 0.1% Tween-20 in PBS was applied at room temperature for 1 h. The primary antibodies (Galectin-3, Invitrogen, MA 1940; RUNX2, Invitrogen, PA582787) diluted in blocking buffer were added and incubated overnight at 4°C. The secondary antibodies (Alexa Fluor 594-labeled Goat Anti-Rabbit IgG and Alexa Fluor 488-labeled Goat Anti-Mouse IgG, Invitrogen) diluted in PBS were then incubated with the cells for 2 h at room temperature. DAPI staining was performed for 5 min, followed by mounting with an anti-fluorescence quenching mounting solution. A laser confocal microscopy was used to capture images of the cells using wavelengths of 405 nm, 488 nm, and 561 nm, and the zeta stack function. Although quercetin possesses intrinsic fluorescence that can be exploited to track its up-take and intracellular distribution, this property does not invalidate the test considering the incubation time used, the nutraceutic degradation and metabolization processes (Zhu et al., 2017; Ma et al., 2018; Cao et al., 2020).

### 2.9 Statistical analysis

The data analysis was performed using GraphPad Prism version 8.0 software (GraphPad Software, San Diego, CA, United States). Data are presented as mean ± SD and analyzed using a one-way ANOVA analysis of variance, followed by the Dunnett test for multiple comparisons. A *p*-value ≤0.05 was considered statistically significant.

## 3 Results

### 3.1 Cytotoxicity of natural extracts

**FIGURE 1:**
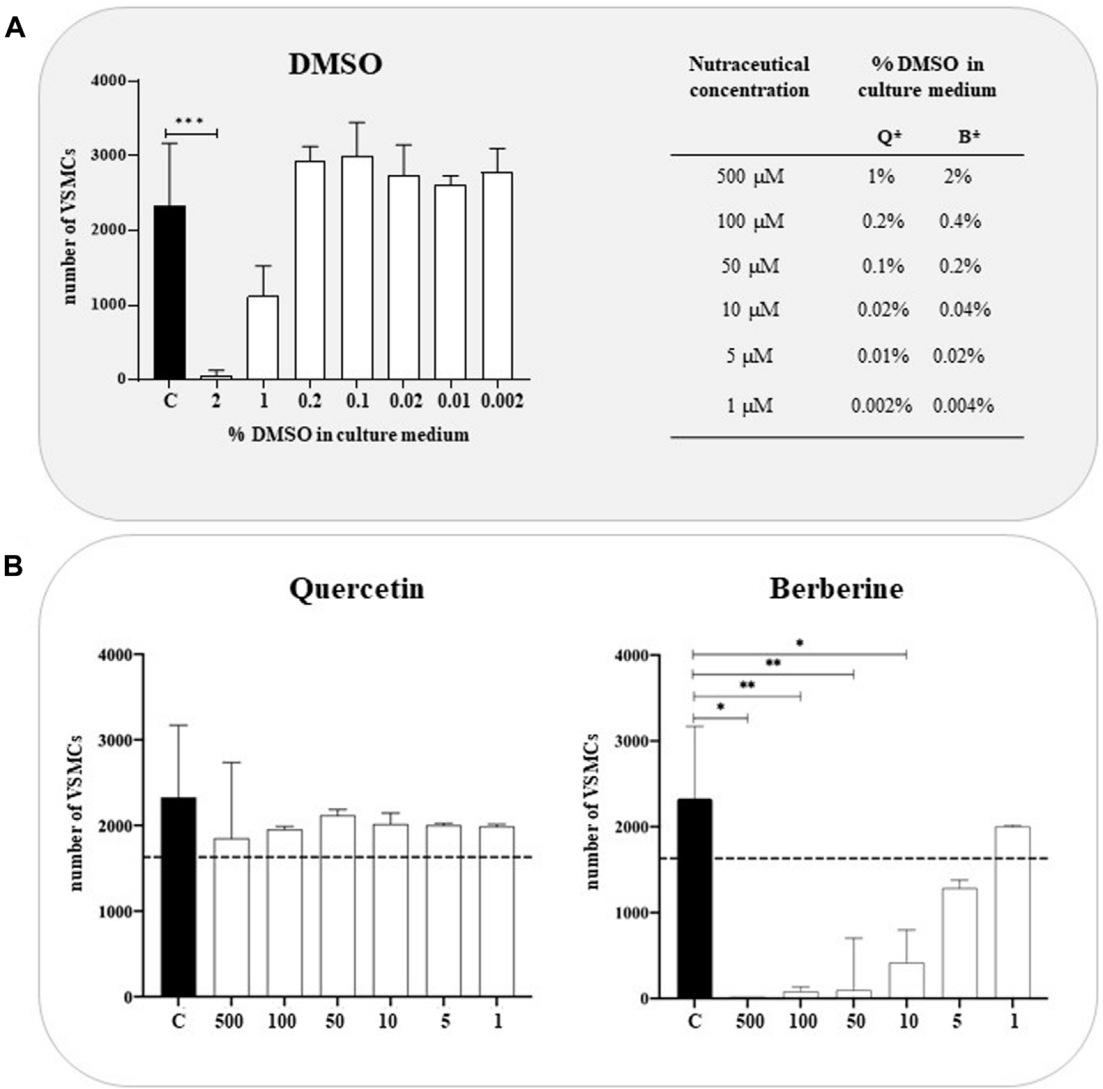
Cytotoxicity of Quercetin and Berberine extracts tested on VSMCs **(A)**. VSMC viability in DMSO-treated cells; C refers to control cells (VSMC grown in medium 231). *Q = Quercetin, B= Berberine **(B)**. VSMC viability following treatment with Quercetin and Berberine extracts in a 1–500 µM concentration range. Data represent the mean of 3 independent experiments. The dotted line corresponds to 70% of cell viability according to EN ISO 10993-5. Statistical analysis was performed with one-way ANOVA and Dunnett multiple comparison test; *p*-value ≤0.05 was considered significant.

A cell viability assay was performed in a concentration range of 1µM and 500 µM of natural extracts to assess any potential cytotoxic effects. The viability of VSMCs, treated with Quercetin and Berberine extracts, was reported in Figure 1. For each concentration tested, an equal amount of DMSO diluted in a culture medium was used as additional control (Figure 1A). As expected, DMSO didn’t affect the cell viability, except at 2%, representing the amount contained in Berberine 500 µM. As reported in Figure 1B, the cytotoxicity of Quercetin was globally very low compared to non-treated cells, with a decrease in cell viability at 500 µM probably related to the presence of 1% DMSO. Interestingly, Berberine extract exhibited marked cytotoxic effects for all the concentrations tested except 1 µM treatment.

### 3.2 Anti-calcifying properties of natural extracts

**FIGURE 2:**
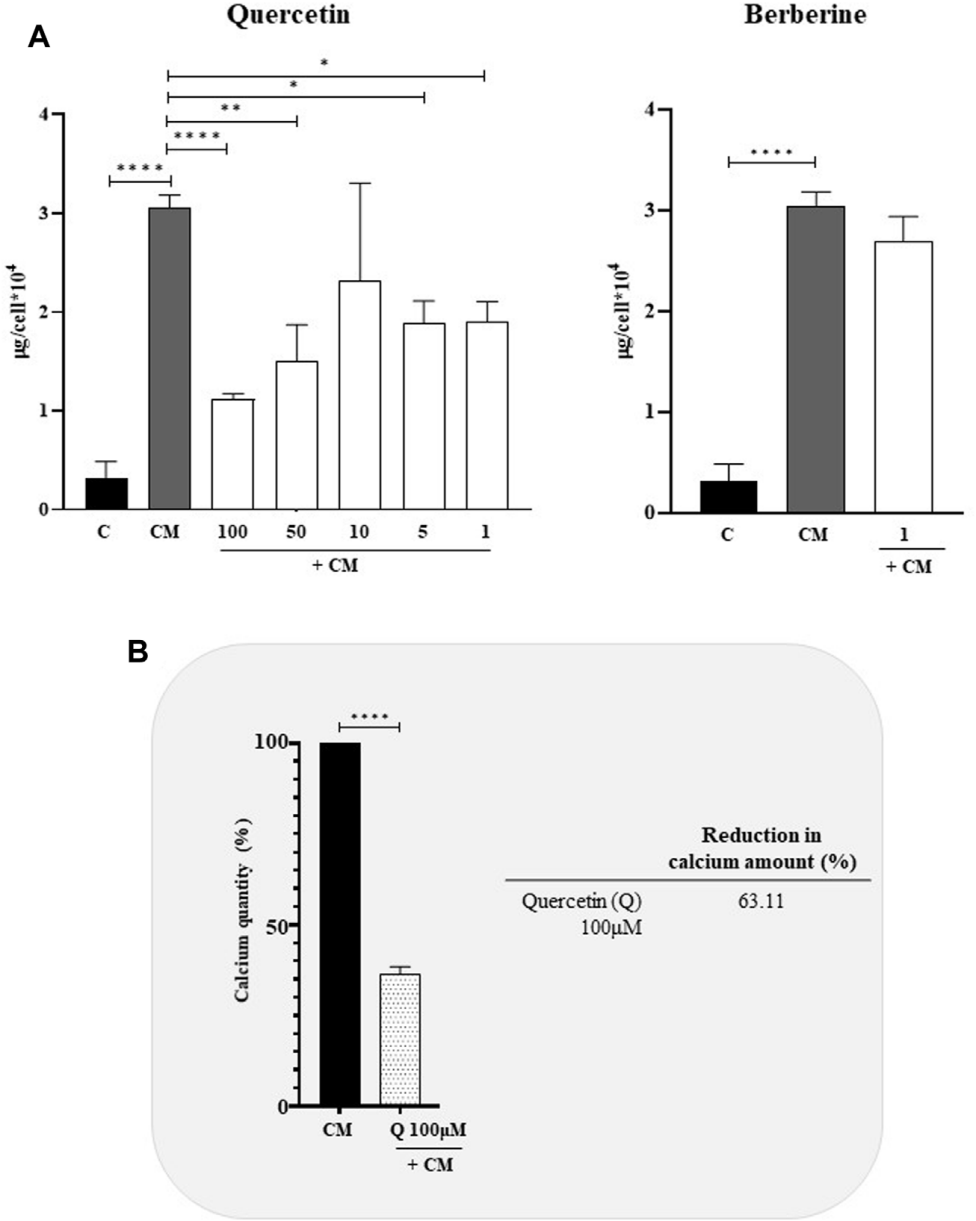
Anti-calcifying activity of Quercetin and Berberine extracts in calcified VSMCs **(A)**. Intracellular calcium content in VSMCs cultured in calcifying medium and treated with different concentrations (μM) of natural extracts **(B)**. Quercetin concentration has shown the highest anti-calcifying ability in calcified VSMCs C: control cells (VSMC grown in medium 231); CM: VSMCs grown in the calcifying medium. Data represent the mean of 3 independent experiments. Statistical analysis was performed with one-way ANOVA and Dunnett multiple comparison test; *p*-value ≤0.05 was considered significant.

To evaluate the anti-calcifying properties of natural extracts, VSMCs cultured in calcifying media were treated with different concentration of Quercetin (1–100 μM) and Berberine (1 μM) and, the intracellular calcium was quantified (Figure 2). Globally, Quercetin reduced the intracellular calcium amount at each concentration tested (Figure 2A), although the greatest reduction (63.11%) was observed at 100 µM concentration (Figure 2B). Concerning Berberine, no significant decrease in intracellular calcium quantity compared to calcified VSMCs was detected at 1 μM, the only concentration tested, being all the other toxic for the cells (Figure 2A).

### 3.3 Anti-calcifying properties of quercetin: Morphological evidences

**FIGURE 3:**
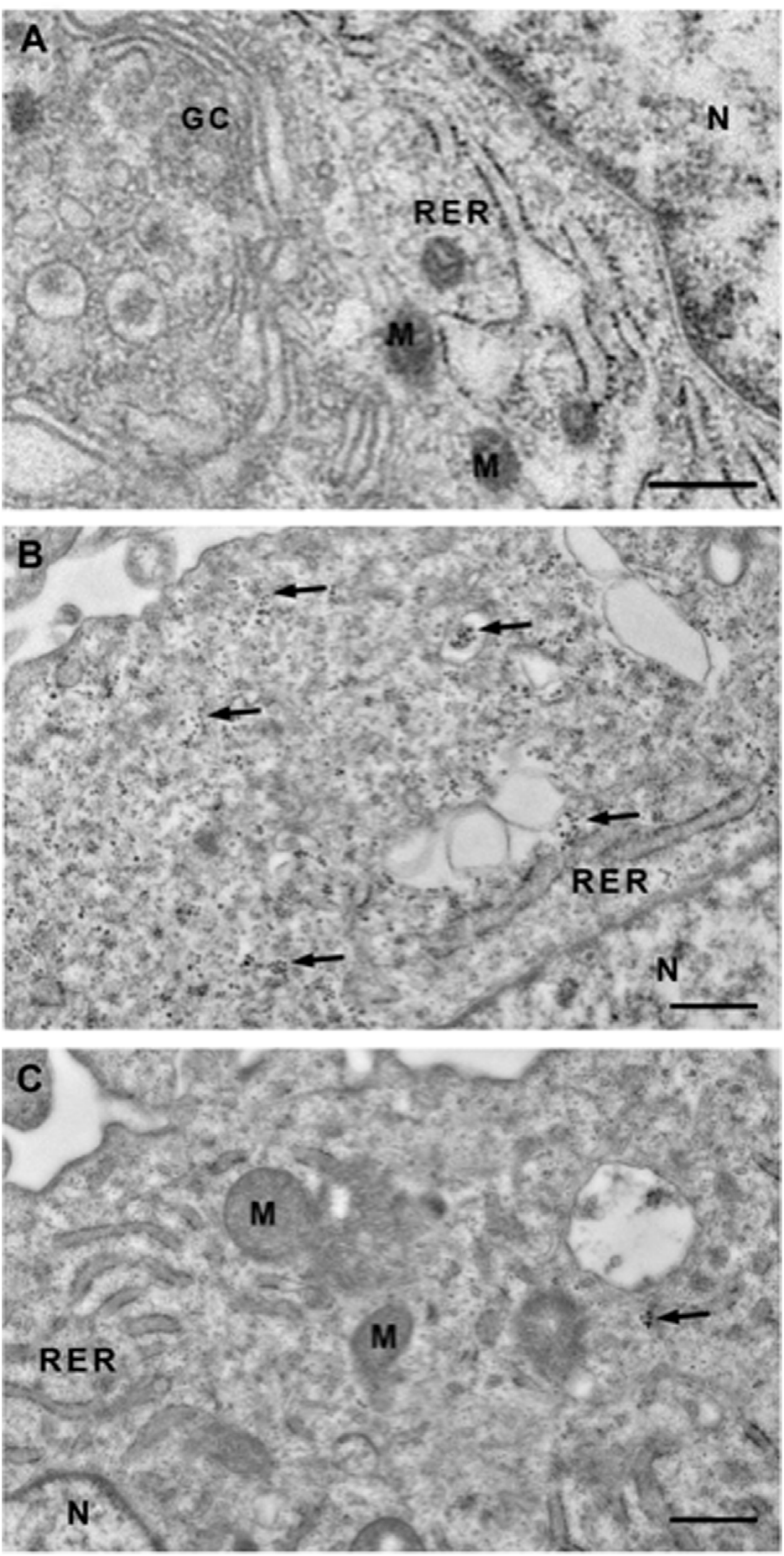
Transmission electron microscopy (TEM) of VSMCs. Representative TEM images of control VSMCs cultured for 7 days in medium 231 **(A)**, in calcifying medium **(B)** and in calcifying medium supplemented with Quercetin extract 100 μM **(C)**. In B, clusters of microcrystals are scattered throughout the cytoplasm (see arrows), sometimes inside vacuoles, and absent in the nucleus. When VSMCs are treated with Quercetin, the cytoplasm is predominantly devoid of clusters. GC: Golgi Complex; RER: Rough Endoplasmic Reticulum; N: Nucleus; M: Mitochondrion. Scale Bar: 500 nm.

Since Quercetin was the most effective natural extract to reduce the intracellular calcium amount, calcified VSMCs were treated with 100 μM Quercetin and processed cell pellets for Transmission Electron Microscopy (TEM) observations to confirm the anti-calcifying properties and verify the intracellular localization (Figure 3). As reported in Figure 3B, TEM highlighted intracellular calcium deposits as microcalcifications (see arrows) located in the cytoplasm and inside vesicles, such deposits were lacking in control cells (Figure 3A). Interestingly, the concomitant treatment with Quercetin extract reduced the amount of calcification, as shown in Figure 3C.

**FIGURE 4:**
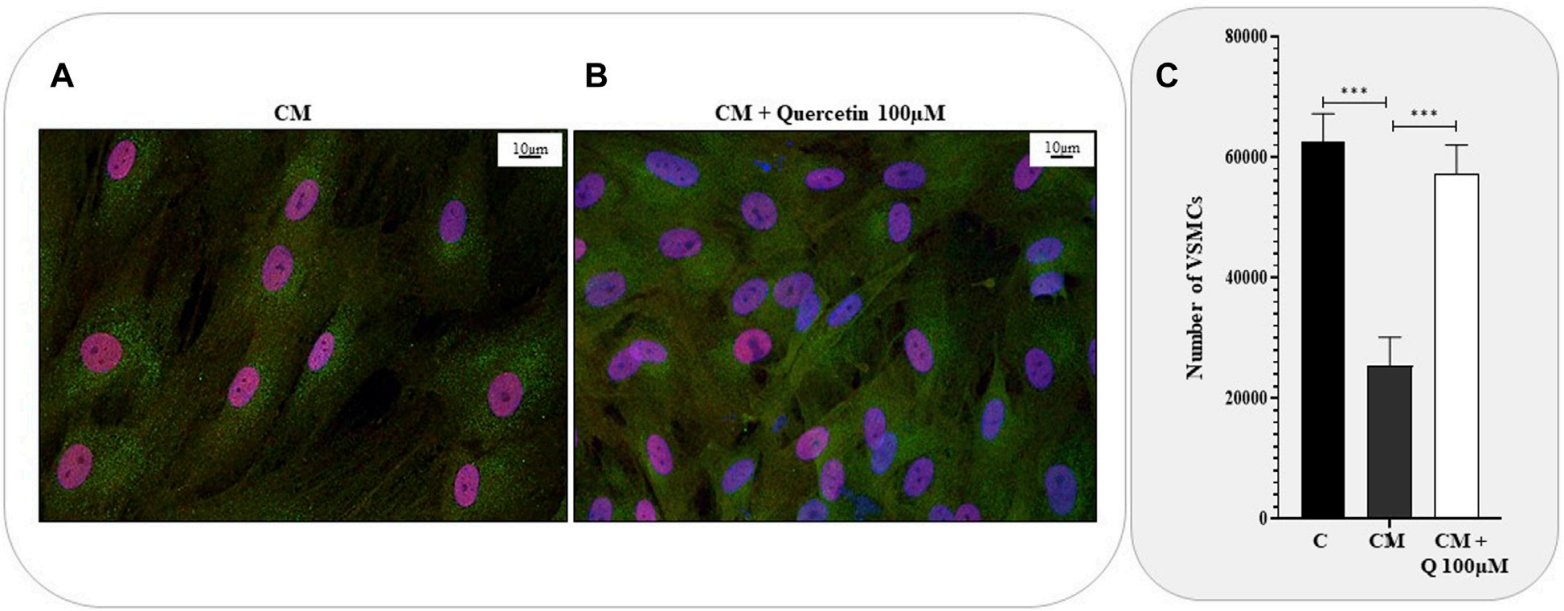
Anti-calcifying properties of Quercetin extract. Representative confocal microscopy images showing the expression of Galectin-3 (green fluorescence) and RUNX2 (red fluorescence) in VSMCs treated with calcifying medium **(A)** and with the concomitant treatment with Quercetin extract **(B)**. Cell nuclei were stained with DAPI (blue fluorescence). Scale bar is 10 μm **(C)**. VSMC viability expressed as number of cells C: control cells (VSMC grown in medium 231); CM: VSMCs grown in the calcifying medium; CM + Q100µM: VSMCs grown in the calcifying medium supplemented with Quercetin extract (Q) at the indicated concentration. Data represent the mean of 3 independent experiments. Statistical analysis was performed with one-way ANOVA and Dunnett multiple comparison test; *p*-value ≤0.05 was considered significant.

To further investigate the effects of Quercetin extract on VSMC phenotype, we also performed immunofluorescence anaylses by confocal microscopy. VSMCs were cultured in calcifyng medium, treated with Quercetin, fixed and eventually incubated with anti-RUNX2 and anti-Galectin-3 antibodies. (Figure 4). Galectin-3 and RUNX-2 are well-known markers of VSMCs in their activated and calcified phenotype. If Galectin-3 is equivalently expressed in calcified (Figure 4A) and Quercetin-treated VSMCs (Figure 4B), on the other hand, variations were observed for RUNX2, which is localised inside the nucleous. Indeed, in calcified VSMCs, RUNX-2 nuclear expression is evident (in every nucleus it co-localizes with DAPI). In Quercetin-treated VSMCs some nuclei still maintain the positivity for the red signal, but others show only the blue fluorescence for DAPI. Moreover, when calcified VSMCs were treated with Quercetin extract, an increased number of cells was noticed. This observation was confirmed by the viability assay (Figure 4C), which showed a significant increase in cell number when VSMCs were treated with Quercetin extract compared to those quantified in calcified VSMCs.

### 3.4 Anti-inflammatory activity of quercetin extract

**FIGURE 5:**
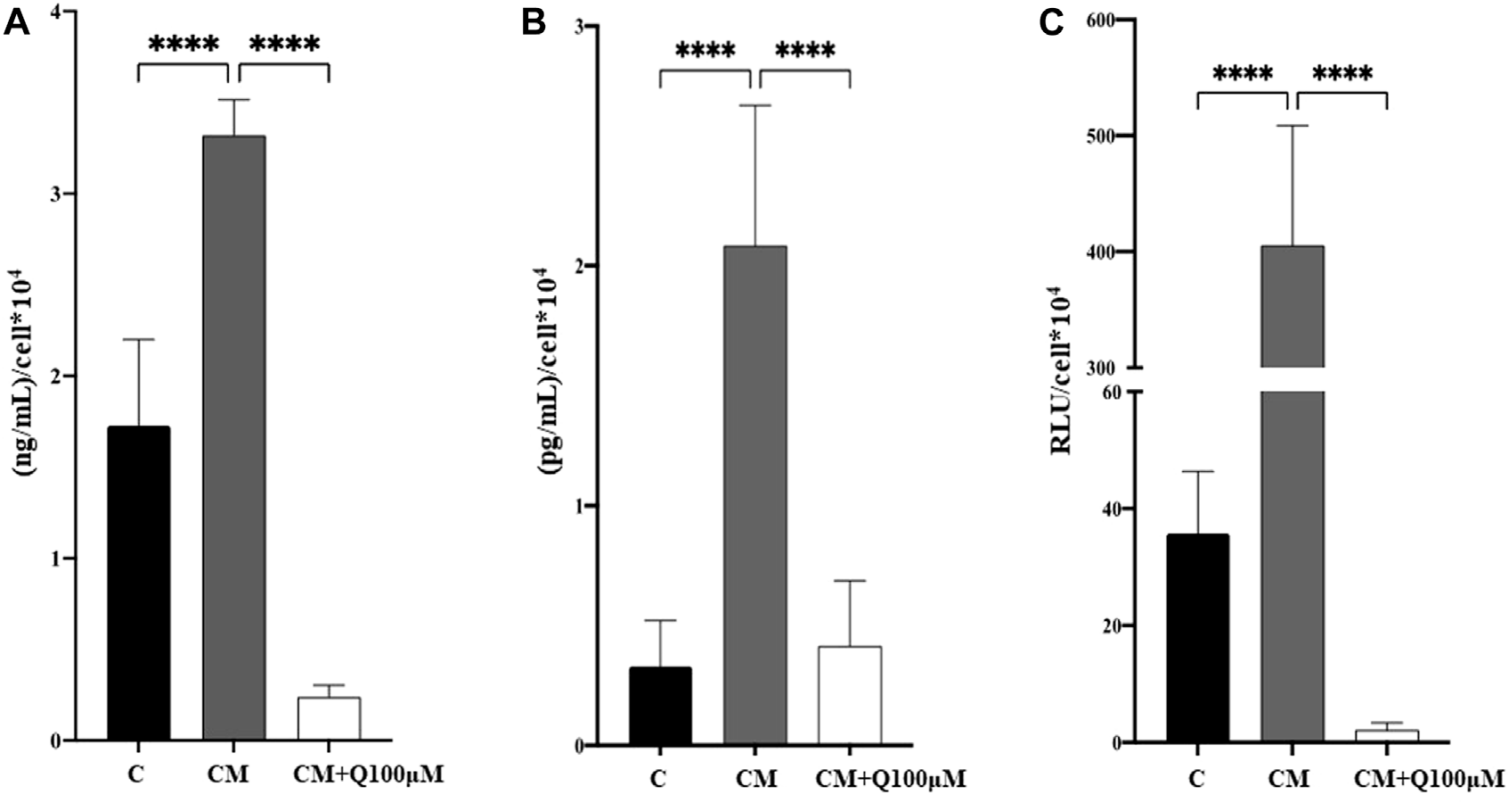
Analysis of the anti-inflammatory properties of Quercetin extract. The amount of IL-6 **(A)**, TNF-α **(B)**, and caspase-1 activity **(C)** was quantified in VSMC culture media. C: control cells (VSMC grown in medium 231); CM: VSMCs grown in the calcifying medium; CM + Q100µM: VSMCs grown in the calcifying medium supplemented with Quercetin extract at the concentration indicated. Data represent the mean of 3 independent experiments. Statistical analysis was performed with one-way ANOVA and Dunnett multiple comparison test; *p*-value ≤0.05 was considered significant.

It has already been demonstrated that increased levels of pro-inflammatory cytokines, such as IL-6, TNF-α and Interleukin-1β (IL-1β) drive the VSMC phenotypic switch, thus triggering VC and its pathological progression. Starting from this evidence, that markers were quantified in VSMC culture media (Figure 5). As expected, calcified VSMCs exhibited a significant increase in inflammatory mediators IL-6 (Figure 5A), TNF-α (Figure 5B), and caspase-1 (Figure 5C) compared to control cells. The concomitant treatment with Quercetin 100 µM significantly counteracted the pro-inflammatory environment associated with VSMC calcification by reducing IL-6, TNF-α, and caspase-1 quantity approximately by 93%, 80%, and 99%, respectively.

## 4 Discussion

**FIGURE 6:**
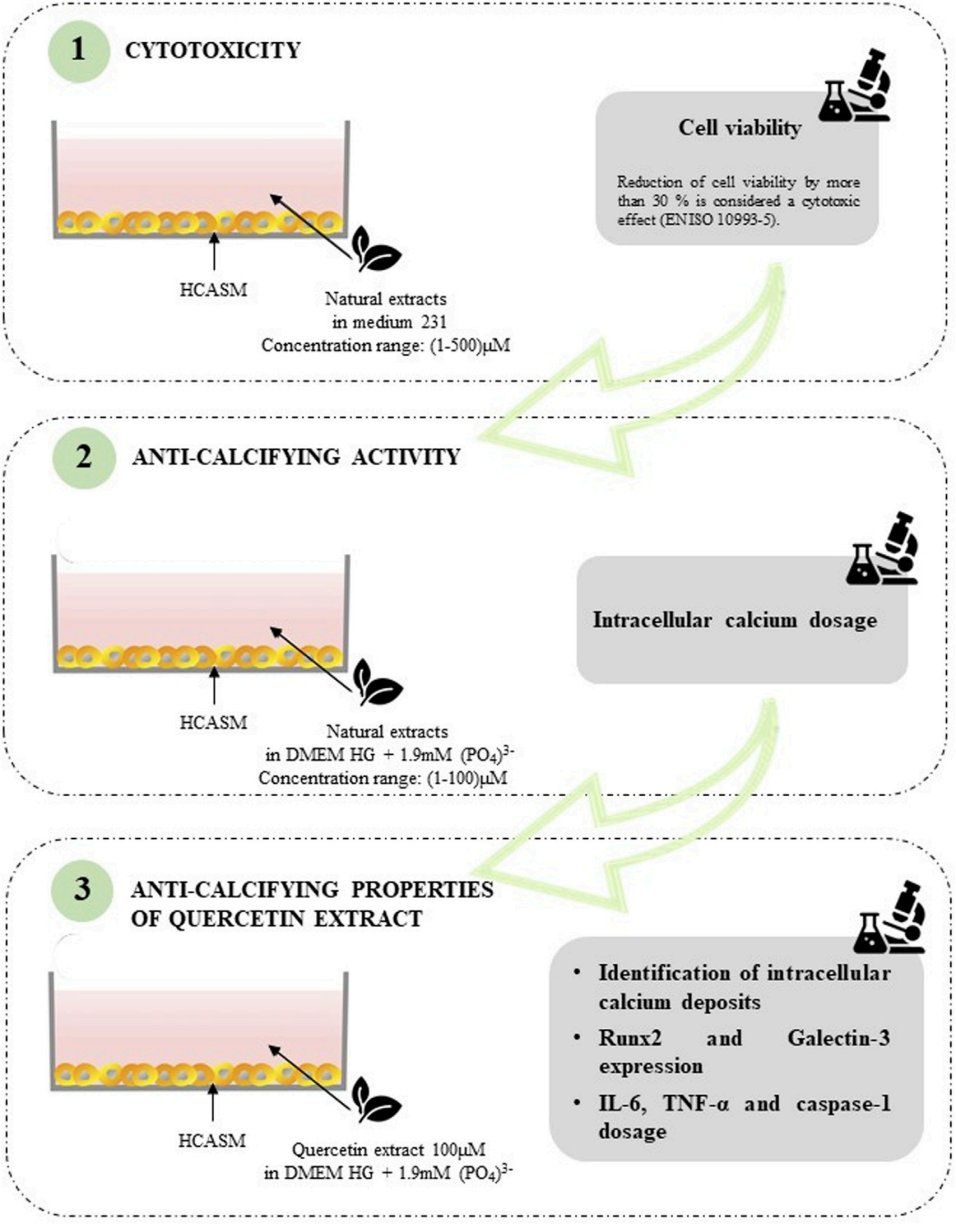
Schematic overview of the experimental workflow.

VC is a pathological condition characterized by the deposition of calcium-phosphate crystals in the vascular system, which occurs in the intimal and medial layers of the vessel wall (Lee et al., 2020). Effective therapies for VC are scant, thus, extensive research efforts have been dedicated to discover new potential anticalcifying drugs. In recent years, natural dietary compounds have emerged as useful candidates in VC treatment (Chao et al., 2019; Ai et al., 2021). In the present study, we tested the anti-calcifying ability of two different natural extracts containing Quercetin (purity of 98.1%) and Berberine (purity of 97.2%) using an *in vitro* model of calcified VSMCs. A graphical overview of the experimental workflow is reported in Figure 6. We first evaluated the cytotoxic potential of nutraceuticals in a concentration range of 1 µM–500µM, showing overall low cytotoxicity, except for Berberine, which induced a marked reduction in cell viability. Quercetin was effective in reducing the intracellular calcium quantity by 63%. This finding was confirmed by TEM analysis. Indeed, VSMCs treated with a calcifying medium for 7 days exhibited micro-calcifications in the cytoplasm and inside vesicles, greatly reduced following Quercetin treatment. Intracellular calcium deposition is a cellular active process of VSMCs undergoing the phenotype change towards an activated osteogenic phenotype (Speer et al., 2009). Published data have correlated this phenotypic switch to the increased expression of several markers, including RUNX2 (Lin et al., 2016; Cobb et al., 2021) and Galectin-3 (Tian et al., 2017; 2021). RUNX2 is a member of the transcription factor family that has been found upregulated in calcified VSMCs, and whose knockout reduced the expression of its downstream osteogenic targets Osterix, Osteocalcin, and Bone sialoprotein, thus attenuating calcification (Sun et al., 2012; Lin et al., 2015; Lin et al., 2016; Cobb et al., 2021). Like RUNX2, Galectin-3-deficient VSMCs exhibited defective expression of osteogenic transcription factors and disorganized mineralization (Menini et al., 2013; Tian et al., 2015). The analysis of these effectors with confocal microscopy confirmed the nuclear presence of RUNX2 and a detectable signal for Galectin-3 in calcified VSMCs. In Quercetin-treated VSMCs, we detected a marked reduction in RUNX2 expression, but no noticeable changes in Galectin-3 expression. Interestingly, cell number increases with Quercetin treatment and the viability assay confirmed this observation, showing an increase in Quercetin-treated VSMCs compared to calcified ones, albeit slightly lower than the viability of control cells, indicating a healthy and proliferative cell state. It has already been demonstrated that inflammation is a key condition that drives the VSMC phenotypic switch, thus triggering VC and its pathological progression (Lee et al., 2021). For example, IL-6 mediates VSMC mineralization through the expression of heat shock protein 70 (HSP70), BMP2, Tissue Non-Specific Alkaline Phosphatase (TNAP), and osteopontin (OPN) (Yao et al., 2009; Sun et al., 2017). Of interest, Zickler and colleagues demonstrated that TNF-α regulates IL-6 secretion through AP-1/c-FOS signaling and promotes VSMC phenotypic transition through increased TNAP activity (Zickler et al., 2018). TNF-α also promoted VSMC apoptosis and the accumulation of apoptotic bodies that promote the pathological deposition of microcalcifications (Proudfoot et al., 2000; Aikawa et al., 2007; Shanahan, 2007). Interleukin-1β (IL-1β) is an important pro-inflammatory mediator synthesized as a biologically inactive polypeptide and processed by caspase-1 to generate the pro-inflammatory cytokine (Sutterwala et al., 2006). Interestingly, IL-1β upregulation has been observed in calcified VSMCs, both *in vitro* and *in vivo* studies (Wen et al., 2013; Awan et al., 2016; Ceneri et al., 2017; Shobeiri and Bendeck, 2017). According to this evidence, we quantified IL-6 and TNF-α levels, and caspase-1 activity in calcified VSMC culture media both in the presence and absence of Quercetin extract. Our data confirmed the increase of inflammatory mediators IL-6, TNF-α, and caspase-1 during cell calcification compared to the amount quantified in controls. Interestingly, in Quercetin-treated VSMCs, we detected a significant reduction in marker expression (greater than 80%) compared to the levels quantified in VSMCs cultured in calcifying medium. Collectively, our data demonstrated that Quercetin extract was effective in reducing VSMC calcification. A possible explanation for its anti-calcifying properties can be found in the interplay between VSMCs phenotypic switch and inflammation. Indeed, Quercetin treatment reduced the inflammatory response, which is a direct inducer of osteogenic transition, thus attenuating the pathological phenotypic switch. The reduction of RUNX2 expression, a marker for calcified phenotype, would support this hypothesis. Moreover, Quercetin restored VSMC viability, possibly interfering with cell apoptosis which represents a key event in the deposition of calcium crystals. Although quercetin has been largely investigated in several *in vitro* models, the novelty of our study concerns the use of primary HCASMCs as screening platform to assess the potentiality of quercetin in vascular calcification. Indeed, published data derived from *in vitro* model HCASMCs-based are scanty, and the majority were obtained in Coronary Artery-SMCs derived from rat (Cui et al., 2017) and in human/rat aortic-SMCs (Lu et al., 2012; Beazley et al., 2013). In our opinion, these preliminary *in vitro* observations could be the starting point for new investigations on the beneficial effects of Quercetin dietary supplementation against VC.

## Data Availability

The original contributions presented in the study are included in the article/Supplementary material, further inquiries can be directed to the corresponding author.
